# Evaluation of *In Vivo* Antiulcer Activity of Hydro-Methanol Extract and Solvent Fractions of the Stem Bark of *Ficus thonningii* (Moraceae) on Rodent Models

**DOI:** 10.1155/2021/6685395

**Published:** 2021-04-09

**Authors:** Habtalem Adane, Seyfe Asrade Atnafie, Zemene Demelash Kifle, Digambar Ambikar

**Affiliations:** ^1^Pharmaceutical Fund and Supply Agency, Bahir Dar, Ethiopia; ^2^Department of Pharmacology, School of Pharmacy, College of Medicine and Health Sciences, University of Gondar, Gondar, Ethiopia

## Abstract

**Introduction:**

The stem bark of *Ficus thonningii* is used by Ethiopian traditional healers and the community for the treatment of peptic ulcer disease. Thus, the current study was aimed at evaluating the antiulcer effect of hydro-methanol extract and solvent fractions of *F. thonningii*.

**Methods:**

The stem bark of *F. thonningii* was collected and shed dried. Then, the stem bark was extracted by 80% hydro-methanol solvents and dried. The part of the dried hydro-methanol extract was further fractionated with n-hexane, chloroform, and distilled water. Dose-dependent pylorus ligation, curative indomethacin-induced, and time-dependent ethanol-induced ulcer models were evaluated for the hydro-methanol extract and solvent fractions. Statistical analysis was done by using the Statistical Package for the Social Sciences (SPSS) version 24. The analyses were carried out using one-way analysis of variance (ANOVA), followed by Tukey's multiple comparison tests. The result was considered significant when *p* < 0.05.

**Results:**

The extract of *F. thonningii* showed a significant (*p* < 0.001) reduction in total acidity at all the tested doses (100, 200, and 400 mg/kg). All the tested doses of the hydro-methanol extract significantly reduced the gastric volume as compared to the vehicle (NC) (*p* < 0.01). The gastric pH was significantly (*p* < 0.05) increased by 200 and 400 mg/kg. Similarly, 200 mg/kg and 400 mg/kg significantly (*p* < 0.05) lowered gastric ulceration as compared to the NC. The hydro-methanol extract and aqueous fractions of *F. thonningii* at 200 mg/kg showed significant (*p* < 0.05) reduction in the ulcer index on a repeated dose of the hydro-methanol and solvent fractions. Ulcer healing effect on indomethacin-induced ulcer was not significant (*p* > 0.05) for all tested doses of the hydro-methanol extract.

**Conclusion:**

The study demonstrated that the stem bark of *F. thonningii* has a potential antiulcer activity that might be due to antisecretory or cytoprotective effects.

## 1. Introduction

Peptic ulcer disease (PUD) is a destruction in a part of the gastrointestinal mucosa that may pass through the muscularis mucosa. Depending on the site of ulcer, PUD can be gastric ulcer (stomach) or duodenal ulcer (duodenum) [1]. PUD is the most common gastrointestinal disease with a worldwide prevalence of about 40% in developed and 80% in developing countries [2]. Although most studies reported a decrease in the incidence or prevalence of gastrointestinal (GI) disorders over time due to a decrease in *Helicobacter pylori*- (*H. pylori*-) associated cases, peptic ulcer disease remains a common condition [3], as it was reported with a point prevalence of 59.6% in Bangladesh [4] and 33.4% in Brazil [5]. In Ethiopia, PUD is highly associated with *H. pylori* infection, alcohol intake, and age [6]. In Tanzania, gastritis (61.10%), gastroesophageal reflux disease (57%), PUD (24.1%), and gastric cancer (6.7%) are the leading causes of dyspepsia [7]. In Ethiopia, a report in Black Lion Hospital indicated patients with complicated PUD comprised 3.8% of the total major surgical procedures [8] and 6.7% of hospital admission in St. Paulos Hospital [9].

Ethiopia is enriched in both medicinal plant biodiversity and cultural knowledge on the ethnopharmacology of medicinal plants [8, 10], which are an integral part of the variety of cultures over centuries for human and livestock medicines [11]. A number of experimental studies have been done on the relationship between plant extracts and their antiulcer activity on animal models. Among these medicinal plant, *I. batatas* [12], *A. vera* [13], *A. integrifolia* [14], *C. colocynthis* [15], *G. glabraon* [16], *Swietenia mahagoni* [17], and *F. religiosa* [18] have been reported with antiulcer activity.

Different parts of *Ficus thonningii* Blume is extensively used by ethnomedical practitioners for treating various ailments in Africa including analgesic, antimicrobial, antimalarial, antihelminthic, antioxidant, cardioprotective, hypotensive, hypoglycemic effects, infertility, impotence, yellow fever, loss of appetite, diarrhea, stomachache, gastritis, gastric ulcers, colitis, influenza, sore throat, colds, arthritis, rheumatism, and to relieve inflammation [19].

*F. thonningii* is grouped in a family of Moraceae. It mainly distributes in upland forests of tropical and subtropical Africa. *F. thonningii* contains different classes of phytochemicals such as flavonoids, tannins, soluble starches, glycosides, alkaloids, terpenoids, and essential oils on the stem bark, leaves, and roots. Furthermore, orientin, vitexin, resveratrol, resveratrol glucosides, and stilbenes glucosides which have antiulcer activities are also identified [20].

The practice of using plants for the treatment of Peptic ulcer disease is also documented in Ethiopia just like other ailments. The stem bark of *F. thonningii* has been used in the treatment of Peptic ulcer disease in Ethiopian folk-medicine without any scientific proof for safety and efficacy [20, 21]. Thus, this study was aimed at evaluating the antiulcer effect of the hydro-methanol extract and solvent fractions of the stem bark of *F. thonningii* (Moraceae).

## 2. Materials and Methods

### 2.1. Drugs, Chemicals, and Supplies

We have methanol (Reagent Chem. Ltd., India), n-hexane (Bululux Laboratories, India), chloroform (Nice Chemicals Lab, Kerala), diethylether (Bululux Laboratories, India), sodium hydroxide (Central Drug House, India), hydrochloric acid, phenolphthalein (Rankem, India), absolute ethanol (Bululux Laboratories, India), ranitidine (Cadilla Pharmaceuticals, Ethiopia), and misoprostol 200 mcg tablet (Jai Pharma Ltd., India).

### 2.2. Collection of Plant Material

*F. thonningii* plant was identified by a botanist and the stem bark was collected from Gondar area, North West Ethiopia, in January 2019. The plant was authenticated, and the specimens were deposited at the University of Gondar herbarium with a voucher number of HA001.

### 2.3. Experimental Animals

Swiss albino mice (20-30 g) and Wistar rats (150–250 g) of either sex were used in this experiment. The laboratory animals were obtained from the National Veterinary Institute, Ethiopia. Animals were contained in standard cages at room temperature and 12 hours of light and dark cycles, and were acclimatized for one week before the study to the laboratory conditions. The animals were fed with a standard pellet and tap water *ad libitum*. The protocol for use of animals was undertaken as per guidelines for use of laboratory animals [22].

### 2.4. Preparation of Hydro-Methanolic Stem Bark Extract

*F. thonningii* stem barks were washed with distilled water to remove dirt and dust. The cleaned plant materials were dried at room temperature. The plant materials were grounded into coarse powder by the electrical mill. Then, the coarse powdered plant materials were macerated separately in hydro-methanol solvent for about 72 hours, then the plant materials were filtered via Whatman No. 1 filter paper and remacerated three times with fresh hydro-methanol solvent. The filtrates of each successive maceration were concentrated by using a rotary evaporator (Yamato Rotary evaporator, Japan) adjusted to a temperature of 40°C. Finally, the semidried residues were frozen in the deep freezer and dried using a lyophilizer (Labfreez, China) to entirely remove the remaining solvent [15, 23].

### 2.5. Fractionation of Hydro-Methanol Extract

n-Hexane, chloroform, and water were used as solvents for fractionation. Briefly, distilled water was added into the crude extract of *F. thonningii* and dissolved by using a separatory funnel. Then, n-hexane was added and shaken to the dissolved components. Similarly, on the aqueous layer, an equal volume of chloroform was added to it, then in both cases, the two layers were separated. The subsequent n-hexane and chloroform layers were separated and exposed to evaporation by using a hot air oven (40°C). Then, the dried hydro-methanol extract and solvent fractions of *F. thonningii* were kept separately in a desiccator until used for the experiment [24, 25].

### 2.6. Phytochemical Screening

Preliminary phytochemical screening tests were carried out to determine the major classes of phytochemicals on the hydro-methanol extract of *F. thonningii* stem bark by using different standard test procedures [26, 27].

### 2.7. Acute Toxicity Test

Acute toxicity study was carried out using the guidelines described by the Organization for Economic Cooperation and Development (OECD) guideline No. 425. Single female Swiss albino mice fasted for four hours on the first day of the test then; 2000 mg/kg of the extract was given by oral route using oral gavage and the mice were observed for the manifestation of behavioral and physical changes and special attention was given during the first four hours. Depend on the results from the first mice, the next 4 females' animals were employed and fasted for about four hours and then a single dose of 2000 mg/kg of the extract was given orally and followed firmly in the same manner. The observation was sustained daily for a total of fourteen days [28].

### 2.8. Grouping and Dosing of Animals

#### 2.8.1. Pylorus Ligation-Induced Ulcer Model

The shay et al. model was used with slight modification [14]. Rats were randomly divided into five study groups, each consisting of six animals. Group I was the negative control (NC), which received a vehicle only (distilled water+6% Tween 80). Group II was served as a positive control and rats were pretreated with ranitidine 50 mg/kg for ten days. Groups III, IV, and V were received 100, 200, and 400 mg/kg of hydro-methanolic extracts of *F. thonningii*.

Rats were fasted for 24 hours before the study but had free access to water till the last 4 hours. After 1 hour of the last drug treatment, animals were anesthetized with diethyl ether and the abdomen was opened by a small midline incision below the xiphoid process. The pyloric portion of the stomach was lifted out and ligated carefully to avoid traction to the pylorus or damage to the blood supply of gastric mucosa. The stomach was replaced carefully and the abdominal wall was closed by interrupted sutures. After four hours of pyloric ligation, rats were sacrificed by inhalational anesthetic ether. The abdomen was opened, the cardiac end of the stomach was dissected out and the content was drained into a test tube. The gastric juice was collected and centrifuged at 1000 rpm for 10 minutes the volume of the supernatant was noted and taken for the determination of total acidity and pH. The stomach mucosa of each animal was washed with saline and running water, labeled, and placed on sodium phosphate-buffered 10% formalin until it was examined for lesions by using a hand lens (10X) and scored accordingly.

#### 2.8.2. Ethanol-Induced Ulcer Model

The ulcer was induced by administering ethanol following the method by Hollander et al. [29], and previous studies to determine the antiulcer effect of repeated and single-dose administration [30, 31]. Animals were randomly assigned to 10 groups each consisting of six animals. Group I and VI received the vehicle (distilled water+4% Tween 80 for single and repeated dose, respectively) and considered as NCs, whereas group II and VII were served as a reference standard and pretreated with misoprostol 5 *μ*g/kg (single and repeated for 10 days, respectively). Groups III, IV, and V were treated with a single dose of 200 mg/kg of hydro-methanol, chloroform, and aqueous fractions, respectively. Group VIII, IX, and X were pretreated with repeated doses of 200 mg/kg of hydro-methanol extract, chloroform, and aqueous fractions of *F. thonningii*, respectively. Animals fasted for 24 hours before the administration of ethanol. All pretreatments of the last dose were given orally 1 hour before the experiment. Gastric ulcer was induced after 60 minutes of *F. thonningii* in 200 mg/kg doses and misoprostol 5 *μ*g/kg treatment by administering ethanol (90% *w*/*v*) at a dose of 1 ml/200 g bodyweight (0.2 ml) to each animal and after 1 hour, animals were sacrificed with spinal dislocation; stomach was incised along the greater curvature and ulceration was scored similarly to pyloric ligation-induced ulcer model [32].

#### 2.8.3. Indomethacin-Induced Ulcer Model

The ulcer was induced with indomethacin at a dose of 18 mg/kg to evaluate the ulcer healing effect of the plant extract which was compared with the NC (vehicle) and positive (misoprostol 5 *μ*g/kg) treated groups. The treatment groups received 100, 200, 400 mg/kg (once daily). The first dose was given 6 hours after induction of ulcer with indomethacin (18 mg/kg). Four days after ulcer induction, analysis has been done [33].

### 2.9. Parameters for the Evaluation of an Antiulcer Activity

#### 2.9.1. Macroscopic Evaluation of the Stomach

The stomach was opened along the greater curvature, rinsed with saline and running water to remove gastric contents and blood clots; then, the mucosa of each animal was labeled and placed on sodium phosphate-buffered 10% formalin until it was examined by a 10x magnifier lens to assess the formation of ulcers. The numbers of ulcers were counted. The Kulkarni method was used for scoring the ulcer as follows: normal colored stomach (0), red coloration (0.5), spot ulcer (1), hemorrhagic streak (1.5), deep Ulcers (2), and perforation (3).

The mean ulcer score (US) for each animal was expressed as the ulcer index (UI). UI was measured by using following formula: UI = UN + US + UP × 10^−1^, where UI is the ulcer index, UN is the average number of ulcers per animal, US is the average number of severity scores, and UP is the percentage of animals with ulcers [34].

#### 2.9.2. Determination of pH and Gastric Volume

An aliquot of gastric juice was taken and the pH of the solution was measured using a pH meter based on the method of Tan [17]. The volume of gastric juice of each animal was measured after centrifugation with 1000 rpm for 10 minutes and analyzed since it is one parameter for the study of the antisecretory effect of the plant extract.

#### 2.9.3. Determination of Total Acidity

An aliquot of 1 ml gastric juice diluted with 9 ml of distilled water was taken and two drops of phenolphthalein indicator were added. Then, it was titrated with 0.01 N NaOH until a permanent pink color was observed. Based on the volume of 0.01 N NaOH consumed, the total acidity was expressed as mEq/l by the following formula [17]:

(1) $$\begin{array}{c} \text{Total Acidity}=\text{Vol}.\text{of NaOH}\times \text{N}\times 100\text{ mEq}/\text{l}/0.1. \end{array}$$

### 2.10. Data Analysis

SPSS version 20 was used for data entry, coding, cleaning, and analysis of results. The result was expressed as the mean ± SEM for each parameter. Statistical differences were evaluated using a one-way ANOVA followed by post hoc Tukey's multiple comparison tests. Results were considered to be statistically significant at (*p* < 0.05).

## 3. Results

### 3.1. Percentage Yields and Preliminary Phytochemical Screening

The percentage yields of the hydro-methanol, chloroform, and aqueous fraction are mentioned in Table 1. However, we do not find sufficient amount of n-hexane fraction for further activity test.

Secondary metabolites like terpenoids, saponins, alkaloids, tannins, anthraquinones, glycosides, phenolic compounds, and flavonoids were found in the hydro-methanol extract of stem bark of *F. thonningii* (Table 2).

### 3.2. Effects of Hydro-Methanol Extract of *F. thonningii* on Pylorus Ligation-Induced Ulcer

All the tested doses of the hydro-methanol extract at 100 mg/kg (*p* < 0.01), 200 mg/kg (*p* < 0.001), and 400 mg/kg (*p* < 0.001) significantly reduced the gastric volume as compared to the NC. Similarly, the standard drug (ranitidine 50 mg/kg) significantly (*p* < 0.001) reduced the gastric volume as compared to the NC. In pyloric ligation model, percentage reduction of gastric volume was found to be 55.8%, 63.3%, 63.4%, and 89.8%, in 100 mg/kg, 200 mg/kg, 400 mg/kg, and ranitidine 50 mg/kg, respectively.

In the present study, all the tested doses and the standard drug (ranitidine 50 mg/kg) significantly reduced the gastric acidity as compared to the NC (*p* < 0.001). Regarding the gastric pH, only 200 mg/kg and 400 mg/kg of *F. thonningii* hydro-methanol extract, showed a significant increment in gastric pH as compared to the NC (*p* < 0.05). In pyloric ligation model, percentage reduction of gastric acidity was found to be 41.9%, 54.8%, 56.4%, and 58.1%, in 100 mg/kg, 200 mg/kg, 400 mg/kg, and 50 mg/kg ranitidine, respectively.

**Figure 1:**
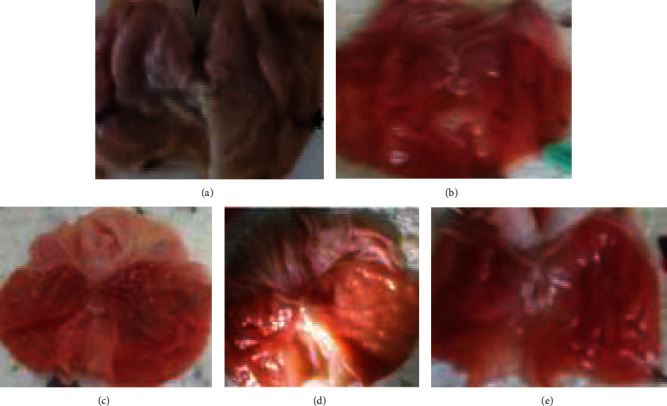
Effect of *F. thonningii* on pylorus ligated rat gastric mucosa pretreated for 10 days: (a) NC: deep ulcer; (b) CE100: deep ulcer, hemorrhagic streak; (c) CE200: red coloration, (d) CE400: red coloration; (e) R50: red coloration. NC = vehicle; CE100 = crude extract at 100 mg/kg; CE200 = crude extract at 200 mg/kg; CE400 = crude extract at 400 mg/kg; R50 = ranitidine at 50 mg/kg.

The current study revealed that pyloric ligation produced gastric ulceration in rat stomach, and pretreatment with the stem bark of *F. thonningii* hydro-methanol extract reduced gastric ulceration. The middle (200 mg/kg) and the higher doses (400 mg/kg) of *F. thonningii* hydro-methanol extract had significantly (*p* < 0.05) lowered gastric ulceration as compared to the NC (Figure 1). In pyloric ligation model, percentage reduction of ulceration was found to be 33.6%, 59.07%, and 62.1% in 100 mg/kg, 200 mg/kg, and 400 mg/kg, respectively (Table 3).

### 3.3. Effect of a Single Dose of *F. thonningii* Hydro-Methanol Extract and Solvent Fractions on the Ethanol-Induced Ulcer

**Figure 2:**
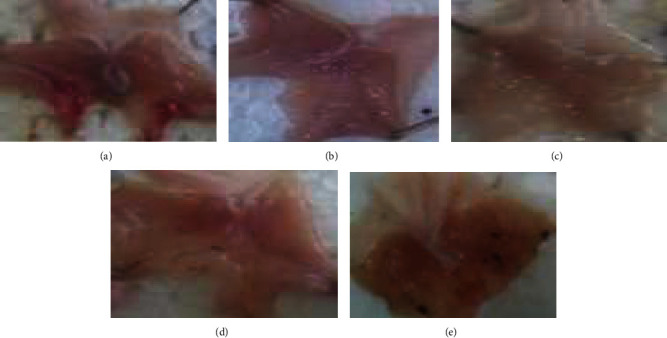
Effect of *F. thonningii* bark extract on ethanol-induced ulcerated gastric mucosa of mice: (a)NC: superficial ulcer and hemorrhagic streak; (b) M5: no ulcer; (c) CE200: red coloration; (d) AF200: red coloration; (e) CF200: spot ulcer, deep ulcer. NC = vehicle; CE200 = crude extract at 200 mg/kg; CF200 = chloroform fraction at 200 mg/kg; AF200 = aqueous fraction at 200 mg/kg; M5 = misoprostol at 5 *μ*g/kg.

The acidified ethanol (90% *w*/*v*) at a dose of 1 ml/200 g bodyweight (0.2 ml) exhibited superficial stomach ulcer and hemorrhagic streak creation in the NC treated mice. However, animals pretreated with the extract, and the standard drug showed a lowered number of ulcers and UI as compared to the NC (Figure 2). There was a difference in the percent reduction in UI and US between the extract-treated group and the NC though failed to reach statistical significance. Nevertheless, misoprostol 5 *μ*g/kg had reduced the UI significantly (*p* < 0.05) as compared to the NC. The percent reduction in the UI was 59.1%, 43.76%, 59.0%, and 73.34% for the hydro-methanol, chloroform fraction, aqueous fraction, and the standard drug, respectively. Likewise, the percent reduction in the US was 60.7%, 30.9%, 30.9%, and 70.24% for the hydro-methanol, chloroform fraction, aqueous fraction, and the standard drug, respectively (Table 4).

### 3.4. Effect of Repeated-Dose of *F. thonningii* Hydro-Methanol Extract and Solvent Fractions on the Ethanol-Induced Ulcer

In the repeated dose acidified ethanol-induced ulcer model, hydro-methanol extract (200 mg/kg), aqueous fraction (200 mg/kg), and misoprostol (5 *μ*g/kg) significantly (*p* < 0.05) prevented ulcer formation as compared to the NC (Figure 2). However, the chloroform fraction (200 mg/kg) did not significantly prevent ulcer formation as compared to the NC. The percent reduction in UI was 71.86%, 39.76%, 70.76%, and 72.96% for the hydro-methanol, chloroform fraction, aqueous fraction, and the misoprostol, respectively. Likewise, the percent reduction in the US was 31.5%, 16.43%, 31.5%, and 54.8% for the hydro-methanol, chloroform fraction, aqueous fraction, and the misoprostol, respectively (Table 5).

### 3.5. Effect of *F. thonningii* Extract on Indomethacin-Induced Ulcer

*F. thonningii* stem bark hydro-methanol extract treatment at a dose of 100, 200, and 400 mg/kg bodyweight did not significantly heal ulcers after 4 days of treatment as compared to the NC. However, misoprostol (5 *μ*g/kg) significantly healed ulcers after 4 days of treatment as compared to the NC (*p* < 0.05). The percent reduction in UI was 55.0%, 61.2%, 63.4%, and 100% for the hydro-methanol extract at 100 mg/kg, 200 mg/kg, 400 mg/kg, and misoprostol 5 *μ*g/kg, respectively. Likewise, the percent reduction in US was 44.6%, 61.5%, 75.4%, and 100% for the hydro-methanol extract at 100 mg/kg, 200 mg/kg, 400 mg/kg, and misoprostol 5 *μ*g/kg, respectively (Table 6).

## 4. Discussion

This study has been done to evaluate the antiulcer effect of *F. thonningii* stem bark considering the global prevalence of the disease and the little attention given to search novel antiulcer agent. The 80% hydro-methanolic solvent is the most preferred one to dissolve both polar and nonpolar phytochemicals since many organic substances are not extracted from aqueous solvents only [35].

In the present study, the preliminary phytochemical screening result revealed the presence of tannins, saponins, anthraquinones, terpenoids, phenolic compounds, flavonoids, and cardiac glycosides. This finding is coherent with report of other Pharmacological research conducted in Zimbabwe [20]. However, the phytochemical result of this study was in contrast with the taxonomical report done in Nigeria [36]. The difference could be due to the solvents used, soil or climatic factors, and the aim of the research. The methanolic extract of stem bark of *F. sycomorus* which is grouped in the same family (Moraceae), had the above metabolites and absence of plant steroids [26].

The above phytochemicals in the study plant have antiulcer activity especially phenolic compounds and flavonoids that possess ideal structural chemistry for free radical scavenging and antioxidative properties [37]. These secondary metabolites when combined with endogenous gastric peroxidases can reduce free radical accumulation and prevent oxidative mucosal damage. The effect may arise from their high reactivity as electron donors. Saponins have antiulcer action by mediating the formation of mucous [38]. Tropane alkaloids (anisodamine and anisodine) which are analogs of atropine block the muscarinic activity of acetylcholine and show antisecretory effect. Quinolizidine alkaloids (matrine, oxymatrine) and indole alkaloids (nigakinone and methylnigakinone) have reported antiulcer effects by decreasing the gastric acid/pepsin secretions and increase the protection of the mucous membrane [39]. Flavonoids can also increase mucosal prostaglandin, decrease histamine secretion by the inhibition of histidine decarboxylase, improve mucus secretion, and inhibition of *H. pylori* growth [40]. The current finding revealed that the study plant was rich in the secondary phytochemicals and the antiulcer effect might be due to the presence of one or more of these secondary metabolites.

The models for the present study have been selected based on the multiple pathophysiology of PUD considering central and peripheral factors as well as the actions of prostaglandins on the disease [41]. In the present study, pylorus ligation (surgical), ethanol (chemical), and indomethacin (pharmaceutical) had been selected. Ulcer formation in pyloric ligation model is due to accumulation of gastric juice and pepsin which results in the auto-digestion of gastric mucosa [42]. Physical stress due to distension of stomach intensifies gastric acid secretion. The gastric acid exposed to the unprotected lumen for long time that results in ulcer. This model is simple, reproducible, and highly predictable which never utilizes exogenous ulcerogenic and suitable for the evaluation of antiulcer drugs with antisecretory mechanisms [43]. The present study showed a significant dose-dependent reduction in the gastric volume by 100 mg/kg (*p* < 0.01), 200 mg/kg (*p* < 0.001), and 400 mg/kg (*p* < 0.001) hydro-methanol extract as compared with the NC. This finding is comparable with the standard drug (ranitidine 50 mg/kg). The percent reduction in gastric volume by the three doses of the extract and the standard drug were 55.8%, 67.5%, 63.4%, and 89.8%, respectively, for 100 mg/kg, 200 mg/kg, 400 mg/kg, and 50 mg/kg of ranitidine. The UI in the NC was almost similar to other studies of the same procedure [15, 44], indicating that the study was done according to the standard procedures. However, the gastric juice volume was lower among the extract-treated groups (5.91 ml versus 8.1 ml) this deference may be due to the time of administration (1 hour versus 45 minutes) which is related to the onset of action of the phytochemicals.

All three doses of the hydro-methanol extract (100, 200, and 400 mg/kg) significantly (*p* < 0.001) decreased the total acidity. The study result confirmed the previous literature reports as even complete elimination of acid secretion and the concept of mucosal cytoprotecting [45]. The dose-dependent antiulcer activity was further supported by UI on 200 mg/kg and 400 mg/kg (*p* < 0.05) while 100 mg/kg and ranitidine 50 mg/kg did not have a significant effect on UI (*p* > 0.05) though it reduces acid secretion significantly *p* < 0.001). In this finding, antisecretory drugs or acid-suppressive therapies may not be necessarily effective on the rate of tissue regeneration, as this scenario of ranitidine was reported in previous studies [15]. There was also a significant change in pH (*p* < 0.05) on 200 mg/kg and 400 mg/kg treated groups. This finding is in agreement with a previous study [46]. This effect suggests that the extract's antiulcer effect is mainly due to its antisecretory in complement with cytoprotective effect or mucosal tissue regeneration of the plant due to the presence of the active phytochemicals.

There was no significant reduction of the UI for all fractions in single-dose treated groups (*p* > 0.05) by the ethanol-induced ulcer model. However, repeated doses of the hydro-methanol extract and aqueous fraction at a dose of 200 mg/kg showed a reduction in UI compared to their respective NC which is statistically significant (*p* < 0.05). Similarly, around 63% reduction in UI was reported from *F. arnottiana* methanolic leaf extract at 500 mg/kg for 10 days [47]. The present study also affirms the importance of repeated dose administration reported by previous studies [30, 46]. The study justified the pharmacokinetic concept of the active principle that the plant extracts having active ingredient need a repeated dose administration to attain its therapeutic window and steady-state for ulcer protective effect.

The hydro-methanol extract and aqueous fraction have shown a statistically significant (*p* < 0.05) reduction in UI. However, the chloroform fraction at 200 mg/kg did not significantly (*p* < 0.05) reduce the UI (Table 5). The current finding indicates that the phytochemicals found in *F. thonningii* which have mucosal cytoprotective activity might be solubilized by relatively polar solvents (water, methanol) than nonpolar solvent like chloroform. This antiulcer effect of the aqueous leaf extract of *F. thonningii* is in line with a previous study [48].

Ethanol-induced ulcers in mice are characterized by heavy bleeding since it can cause immediate stasis in the blood flow [48]. Previous studies showed that (50% *w*/*v*) ethanol can induce gastric damage in >90% of test animals and lesions are blackish of varying size parallel to the major axis [49]. Lesions can be inhibited by various drugs like misoprostol. Ethanol can cause turbulences in gastric secretion, alter the permeability of the gastric membrane, and free radical production. Oxygen-derived free radicals have been found in the mechanism of acute ulceration in the gastric mucosa leading to increased intracellular membrane permeability to sodium and water and enormous intracellular increase of calcium that corresponds to the pathogenesis of gastric mucosal injury [50]. Plant principles which prevent ulcer can have cytoprotective effects in general. It was interesting that a similar antiulcer effect was observed by the plant extracts which could be due to the above-mentioned mechanisms.

In the indomethacin-induced ulcer model, all doses of the hydro-methanol extract (100, 200,400 mg/kg) did not have a significant effect (*p* > 0.05). In the ulcer induction protocol, the dose of indomethacin was raised to 18 mg/kg which caused the US of 0.65 ± 0.15; which was lower as compared with the report done in Addis Ababa University (dose 28 mg/kg; US = 1.83 ± 0.17) [49]. A dose-dependent antiulcer activity was recorded in the indomethacin model which indicated the medicinal plants with low potency on ulcer healing effect may need a higher dose to produce full protection against the ulcerogenic effect of NSAIDs. A similar study on the extract of *Dialium guineense* revealed that the antiulcer effect was shown at the highest dose of the extract (750 mg/kg) [51].

Inhibition of prostaglandin synthesis by blocking the enzyme cyclooxygenase (COX) with nonsteroidal anti-inflammatory drugs is the second most common etiologic agent of PUD next to *H. Pylori.* These aggressive factors can increase the susceptibility of gastrointestinal mucosa by luminal irritants, alter the microcirculation that is critical to the pathogenesis of ulceration, and damage the mucus and bicarbonate secretion [52]. The present finding suggested that, the antiulcer activity of *F. thonningii* may not be related to modulation of prostaglandin synthesis or nonselective inhibition of the constitutive enzyme (COX-1) and inflammatory enzyme (COX-2).

The hydro-methanolic extract of *F. thonningii* stem bark did not show any sign of acute toxicity up to 2000 mg/kg. This suggesting that the stem bark of the plant is relatively nontoxic and the same result has been reported on the leaf extract of *F. thonningii* in Nigeria and the fruit was reported as edible in Ethiopia [36, 53, 54].

## 5. Conclusion

The present study demonstrates that the hydro-methanol crude extract and aqueous solvent fraction of the stem bark of *F. thonningii* have an antiulcer effect, which may probably be related to the antisecretory, mucosal protecting, anti-inflammatory, or free radical scavenging activity of the phytochemical constituents reported. This study justifies the traditional claims on folk medicine reported in Ethiopia and Nigeria. The plant extracts also exhibited a good safety profile at 2000 mg/kg dose. Therefore, the aqueous fraction and the hydro-methanol crude extract could represent a new source for the development of a new plant-derived antiulcer agent.

## Figures and Tables

**Table 1:** Percentage yield of *F. thonningii* stem bark extract.

Extract	Yield (g)	Percentage yield
Crude extract	135/1000 g bark	13.5%
Aqueous fraction	12.0/67 g crude	17.91%
Chloroform fraction	5.5/67 g crude	8.2%
n-Hexane fraction	—	Very small

Key: — indicates very small amount to calculate the yield.

**Table 2:** Preliminary phytochemical screening of *F. thonningii* stem bark.

Test	Phytochemical	Result
1	Terpenoids	+
2	Saponins	+
3	Alkaloids	+
4	Tannins	+
5	Plant steroids	-
6	Anthraquinones	+
7	Glycosides	+
8	Phenolic compounds	+
9	Flavonoids	+

Key: + indicates the presence of the constituents; - indicates the absence of the constituents.

**Table 3:** Antiulcer (antisecretory) effect of the crude extract of *F. thonningii* on the pylorus ligated-induced ulcer model.

Treatment	Volume of gastric juice	Reduction of vol (%)	Total acidity	Reduction in acidity (%)	pH	Ulcer index (UI)	Reduction in UI (%)
NC	5.91 ± 0.62	—	51.66 ± 3.07	—	3.16 ± 0.16	15.93 ± 0.79	—
CE100	2.61 ± 0.81^a2^	55.8	30.00 ± 2.58^a3^	41.9	4.50 ± 0.34	10.58 ± 2.14	33.6
CE200	2.16 ± 0.24^a3^	63.3	23.33 ± 2.10^a3^	54.8	5.00 ± 0.61^a1^	6.52 ± 2.74^a1^	59.07
CE400	2.02 ± 0.30^a3^	63.4	22.53 ± 3.07^a3^	56.4	5.33 ± 0.51^a1^	6.04 ± 2.7^a1^	62.1
R50	0.60 ± 0.13^a3^	89.8	21.66 ± 3.07^a3^	58.1	5.17 ± 0.30^a1^	8.20 ± 2.60	48.5

Values are expressed as means ± SEM (*n* = 6). ^1^*p* < 0.05, ^2^*p* < 0.01, and ^3^*p* < 0.001. NC = vehicle; CE100 = crude extract at 100 mg/kg; CE200 = crude extract at 200 mg/kg; CE400 = crude extract at 400 mg/kg; R50 = ranitidine at 50 mg/kg; ^a^ = compared with the negative control group; CEFT: crude extract of *Ficus thonningii*. Statistical comparisons are significant at *p* value < 0.05.

**Table 4:** Effect of hydro-methanol and solvent fractions on ethanol-induced ulcer model (single dose).

Treatment	Ulcer score (US)	Reduction in US (%)	Ulcer index (UI)	Reduction in UI (%)
NC	0.84 ± 0.14	—	14.67 ± 0.99	—
CE200	0.33 ± 0.17	60.7	6.0 ± 2.68	59.1
CF200	0.58 ± 0.24	30.9	8.25 ± 2.61	43.76
AF200	0.58 ± 0.30	30.9	6.08 ± 2.72	59.0
M5	0.25 ± 0.17	70.24	3.91 ± 2.47^a1^	73.34

Values are expressed as mean ± SEM (*n* = 6). ^1^*p* < 0.05, ^2^*p* < 0.01, and ^3^*p* < 0.001. NC = vehicle; CE200 = crude extract at 200 mg/kg; CF200 = chloroform fraction at 200 mg/kg; AF200 = aqueous fraction at 200 mg/kg; M5 = misoprostol at 5 *μ*g/kg; ^a^ = compared with NC (95% CI). Statistical comparisons are significant at *p* < 0.05.

**Table 5:** Effect of hydro-methanol and solvent fractions on ethanol-induced ulcer (10 days).

Treatment	Ulcer score (US)	Reduction in US (%)	Ulcer index (UI)	Reduction in UI (%)
NC	0.73 ± 0.08	—	15.39 ± 0.72	—
CE200	0.5 ± 0.31	31.5	4.33 ± 2.74^a1^	71.86
CF200	0.61 ± 0.27	16.43	9.27 ± 2.98	39.76
AF200	0.5 ± 0.31	31.5	4.5 ± 2.8^a1^	70.76
M5	0.33 ± 0.24	54.8	4.16 ± 2.6^a1^	72.96

Values are expressed as mean ± SEM (*n* = 6). ^1^*p* < 0.05, ^2^*p* < 0.01, and ^3^*p* < 0.001. NC = vehicle; CE200 = crude extract at 200 mg/kg; CF200 = chloroform fraction at 200 mg/kg; AF200 = aqueous fraction at 200 mg/kg; M5 = misoprostol at 5 *μ*g/kg; ^a^ = compared with NC. Statistical comparisons are significant at *p* < 0.05.

**Table 6:** Ulcer healing effect of the hydro-methanol extract on indomethacin-induced ulcer (4 days of treatment).

Treatment	Ulcer score (US)	Reduction in US (%)	Ulcer index (UI)	Reduction in UI (%)
NC	0.65 ± 0.15	—	10.45 ± 2.1	—
CE100	0.36 ± 0.10	44.6	4.70 ± 2.50	55.02
CE200	0.25 ± 0.17	61.5	4.06 ± 2.58	61.15
CE400	0.16 ± 0.10	75.4	3.83 ± 2.42	63.35
M5	0.00 ± 0.00^a1^	100	0.00 ± 0.00^a1^	100

Values are expressed as means ± SEM (*n* = 6). ^1^*p* < 0.05, ^2^*p* < 0.01, and ^3^*p* < 0.001. NC = vehicle; CE100 = crude extract at 100 mg/kg; CE200 = crude extract at 200 mg/kg; CE400 = crude extract at 400 mg/kg; M5 = misoprostol at 5 *μ*g/kg; ^a^ = compared with the negative control group. Statistical comparisons are significant at *p* < 0.05.

## Data Availability

The original data used to support the findings of this study have been deposited in the national academic of digital repository of Ethiopia (https://nadre.ethernet.edu.et//record/1806#.X5-iFFgzbIU).
